# Comparison of Propofol or Isoflurane Anesthesia Maintenance, Combined with a Fentanyl–Lidocaine–Ketamine Constant-Rate Infusion in Goats Undergoing Abomasotomy

**DOI:** 10.3390/ani11020492

**Published:** 2021-02-13

**Authors:** Perla I. Velázquez-Delgado, Eduardo Gutierrez-Blanco, Felipe de J. Torres-Acosta, Antonio Ortega-Pacheco, Armando J. Aguilar-Caballero, Brighton T. Dzikiti

**Affiliations:** 1 Department of Animal Health and Preventive Medicine, Autonomous University of Yucatan, 97000 Merida, Mexico; p.ivette.velazquez@gmail.com (P.I.V.-D.); tacosta@correo.uady.mx (F.d.J.T.-A.); opacheco@correo.uady.mx (A.O.-P.); aguilarc@correo.uady.mx (A.J.A.-C.); 2 Department of Large Animal Clinical Science, School of Veterinary Medicine, Ross University, KN0111 Basseterre, Saint Kitts and Nevis; tdzikiti@rossvet.edu.kn

**Keywords:** anesthesia, goat, isoflurane, partial intravenous anesthesia, propofol, total intravenous anesthesia

## Abstract

**Simple Summary:**

General anesthesia in small ruminants is still a challenge under field conditions. Propofol is an injectable short-acting anesthetic used to provide induction and/or anesthesia maintenance. Isoflurane is the inhaled anesthetic more widely used for providing general anesthesia; however, it requires an expensive equipment for its administration, and high doses may produce environmental pollution. Both anesthetics produce dose-related cardiovascular depressant effects. This study aimed to compare the effects of propofol or isoflurane, combined with a constant-rate infusion of fentanyl–lidocaine–ketamine (total [total intravenous anesthesia (TIVA)] and partial intravenous anesthesia [PIVA], respectively) in goats undergoing abomasotomy. Our results showed that both TIVA and PIVA protocols produced a satisfactory quality of anesthesia during surgery, with minimal changes in cardiopulmonary parameters. However, recovery from anesthesia induced by propofol fentanyl–lidocaine–ketamine might be of poor quality.

**Abstract:**

This study aimed to compare, first, the anesthetic and cardiopulmonary effects of propofol or isoflurane anesthetic maintenance in goats receiving a fentanyl–lidocaine–ketamine infusion undergoing abomasotomy and, secondly, to compare the quality of the recovery from anesthesia. Two groups were used: propofol (TIVA) and isoflurane (PIVA). Goats were premedicated with fentanyl (10 μg/kg intravenously [IV]), lidocaine (2 mg/kg, IV), and ketamine (1.5 mg/kg, IV). Anesthesia was induced with propofol and maintenance consisted of fentanyl (10 μg/kg/h, IV), lidocaine (50 μg/kg/min, IV), and ketamine (50 μg/kg/min, IV) as constant-rate infusions (CRIs), combined with either CRI of propofol at initial dose of 0.3 mg/kg/min, IV (TIVA), or isoflurane with initial end-tidal (FE’Iso) concentration of 1.2% partial intravenous anesthesia (PIVA). The mean effective propofol dose for maintenance was 0.44 ± 0.07 mg/kg/min, while the mean FE’Iso was 0.81 ± 0.2%. Higher systolic arterial pressure (SAP) values were observed in total intravenous anesthesia (TIVA) during some time points. Recovery was smooth in PIVA, while restlessness, vocalizations, and paddling were observed in TIVA. Both protocols produced a satisfactory quality of anesthesia during surgery, with minimal impact on cardiopulmonary function. Nevertheless, recovery after anesthesia in TIVA might be of poor quality.

## 1. Introduction

Goats are being increasingly used as a surgical model for a variety of biomedical research applications and need to be subjected to an efficient general anesthesia method [1]. Ordinarily, anesthesia is achieved using injectable anesthetic drugs for induction and maintenance of anesthesia, while inhaled agents are used for maintenance of anesthesia [2].

In the current anesthetic practice, there is no single ideal anesthetic agent; even if some agents have specific advantages, they lack other important ideal properties.

The selection of the best technique for anesthetic maintenance should be based on the intrinsic pharmacological effects of each anesthetic, type of procedure and its duration, on the availability of inhalation anesthetic equipment, on the proficiency in the anesthetic technique, etc. [3,4].

When anesthesia is maintained exclusively with a single inhaled agent (i.e., in the absence of other sedatives or analgesics drugs), high doses of anesthetics are required to achieve a satisfactory anesthetic plane, which increases the likelihood of adverse effects (e.g., dose-related cardiovascular depression) [5]. In addition, the use of a single inhaled agent often requires expensive special equipment and installations for administration, and inhaled agents such as isoflurane, are chlorofluorocarbons, compounds potentially dangerous to the earth’s ozone layer [6], and may contribute to global warming [7].

Balanced anesthetic techniques are often an option to a single anesthetic-based anesthesia. They include the simultaneous usage of multiple drugs in combination, to achieve the four main components of the ideal anesthetic state (amnesia, analgesia, muscle relaxation, and the stability of systems) with as few side effects as possible [3,8].

An alternative to a single agent inhaled anesthesia, is the use of inhaled anesthetics with intravenous drugs (analgesics and/or sedatives), this protocol is known as partial intravenous anesthesia (PIVA) [4,9,10] and leads to an improvement in cardiopulmonary parameters, due to the reduction in the anesthetic requirements of inhaled anesthetic agents to prevent intraoperative awareness and thus reducing its dose-related cardiovascular depressing effects [11,12,13].

General anesthesia can also be maintained using total intravenous anesthesia (TIVA), which is an alternative to inhaled anesthetics [14]. Because of its pharmacokinetic properties (that allow a rapid onset), ultra-short action, and rapid recovery, propofol has proven to be the most suitable agent for TIVA protocols in dogs, cats, horses, calves and goats [14,15]. Comparatively to inhaled anesthesia, injected anesthesia has a lower cost, and requires minimal equipment for delivery and control [4].

Because it lacks pain-relieving effects, isoflurane, similar to propofol, should be combined with analgesic drugs for anesthesia during painful procedures; various drugs have been used for this purpose [1,9,10,14,16,17,18,19,20].

Several studies have reported the effects of analgesic drugs, such as fentanyl, lidocaine, and ketamine, or of the combination of lidocaine and ketamine constant-rate infusions (CRIs), on the anesthetic requirements for isoflurane in dogs and goats [10,21,22,23] and propofol.

The combinations of analgesic drugs with different pharmacologic mechanisms may provide a higher degree of analgesia than each drug administered alone, which may result in a significant decrease in hypnotic agents such as propofol and isoflurane [20,23,24].

Current literature lacks a description of the effects of the combination of fentanyl, lidocaine, and ketamine in goats when co-administered as pre-anesthetics and/or during anesthesia. This description is important because it allows the determination of the sparing and analgesic effects of this combination of agents, and to characterize the post-surgical recovery; good recovery quality is essential for safety and welfare of the patient [4].

This study aimed to compare the anesthetic, cardiopulmonary effects, and recovery of propofol and isoflurane, used for anesthetic maintenance, in goats undergoing abomasotomy receiving a fentanyl–lidocaine–ketamine constant-rate infusion. We hypothesized that anesthesia maintenance with propofol or isoflurane could produce comparable results, with minimal changes in cardiopulmonary parameters and comparable recovery quality.

## 2. Materials and Methods

This study was a prospective, randomized, non-blinded clinical trial. Researchers obtained ethical approval for this study protocol from the Research Ethics committee of the Faculty of Veterinary Medicine and Animal Science of the Autonomous University of Yucatan (Protocol number CB-CCBA-M-2017-001).

### 2.1. Animals

Eighteen mature female, non-pregnant tropical creole goats aged 4.74 ± 2.31 years and weighing 32.51 ± 4.96 kg were used. Goats were healthy according to physical examination, complete blood count, serum biochemical, and fecal analyses. Does were undergoing surgery as a part of a different study where, as in the present study, the unique inclusion criteria were to be healthy, non-pregnant and adult (middle aged) mature goats.

### 2.2. Anesthetic Procedure and Study Design

Food and water were withheld for 24 h before anesthesia. The reference point measurements for heart rate (HR), respiratory rate (Rr), rectal temperature, hemoglobin oxygen saturation (SpO_2_), end-tidal CO_2_ (ET-CO_2_)and oscillometric blood pressure were recorded in individual housing pens 15 min before the beginning of sedation. An appropriately sized cuff (approximately 40% of the circumference of the limb) was placed directly over the right forearm, in alignment to the metacarpal artery of does for blood pressure measurements. An 18-gauge catheter (Introcan^®®^; B-Braun, São Gonçalo, Brazil) was inserted into the left jugular vein for administration of intravenous fluids and drugs.

For sedation, intravenous (IV) fentanyl (10 μg/kg; Fenodid; Pisa Farmaceutica, Guadalajara, Mexico), lidocaine (2 mg/kg; Pisacaina 2%; Pisa Farmaceutica, Guadalajara, Mexico) and ketamine (1.5 mg/kg, Anesket; Pisa Farmaceutica, Guadalajara, Mexico) were sequentially administered over one minute period, respectively. Ten minutes later, the degree of sedation was assessed using a 0–3 scale as follows: 0 = no sedation, 1 = light sedation (the goat lowers its head), 2 = moderate sedation (the goat adopts a sternal position, but can raise its head), 3 = deep sedation (the goat adopts a sternal position and is unable to raise its head). Immediately before induction of anesthesia, the goats were preoxygenated (100% oxygen) for three minutes using a face mask, at a flow rate of 5 L/min.

Once sedated, a 22-gauge catheter (Introcan^®®^; B-Braun, São Gonçalo, Brazil) was inserted into the right auricular artery to facilitate measurement of direct blood pressure and for the collection of arterial blood samples for gas analysis.

General anesthesia was induced IV with propofol (propofol 1%; Fresenius Kabi, Linz, Austria) at an initial dose of 3 mg/kg; additional doses were administered as needed to allow easy orotracheal intubation using an appropriately sized orotracheal cuffed tube. Propofol was manually injected for 30 s. Following endotracheal intubation, goats were connected to a circle re-breathing system (Multiplus MEVD; Royal Medical Co. Ltd., Yeongdeungpo-gu, South Korea) and the oxygen flow rate was initially set at 50 mL/kg/min and the CRIs started. The CRIs of fentanyl at 10 μg/kg/h; ketamine at 50 μg/kg/min and lidocaine at 50 μg/kg/min were started IV at this time.

Goats were allocated to either PIVA or TIVA protocols based on the drugs used for maintenance of general anesthesia, using a computer-generated random number (QuickCalcs GraphPad Software, www.graphpad.com/quickcalcs/randomize1/). Drugs for maintenance of anesthesia were administered as follows:

TIVA protocol: Initial CRIs of propofol at 0.3 mg/kg/min. The propofol infusion rate was adjusted to maintain surgical anesthesia as described below (see section “Determinants of isoflurane vaporizer setting and propofol CRI”).

PIVA protocol: Isoflurane vaporized in oxygen at an initial end-tidal (FE′Iso) of 1.2% and oxygen flow rate of 100 mL·kg^−1^ min for the first 10 min, and 50 mL·kg^−1^ min thereafter. Isoflurane administration rate was adjusted to maintain surgical anesthesia as described below (see section Determinants of isoflurane vaporizer setting and propofol CRI).

For anesthesia maintenance, propofol was administered by a pre-calibrated syringe pump device (Graseby 3400; Graseby Medical, Hertfordshire, UK). Except when propofol was used for induction of anesthesia, drugs for CRIs were diluted to 50 mL with sterile water and administered using a triple-channel infusion pump device (Colleague 3: Baxter, Deerfield, USA); propofol infusion started immediately after completing the administration of the last bolus of propofol for induction of general anesthesia. Hartmann solution (Solution Ht, Pisa, Guadalajara, Mexico) was administered at 5 mL/kg/h throughout anesthesia using an infusion pump device (Colleague 3: Baxter, IN., USA). IV fluids, propofol infusions, and fentanyl–lidocaine–ketamine infusions were simultaneously administered using two 3-way valves connected to the main fluid line.

### 2.3. Cardiopulmonary Variables Measurement

HR and rhythm were observed from a lead II ECG configuration. Systolic arterial pressure (SAP), mean arterial pressure (MAP) and diastolic arterial pressure (DAP) were continuously measured from the right auricular artery using a multiparameter monitor (PM 9000 Vet Mindray, Shenzhen, China), which was connected to a heparinized saline line tube that was attached to an electronic pressure transducer; it was zeroed to barometric pressure, and adjusted to the heart level while the goat was in dorsal recumbence. Hemoglobin oxygen saturation (SpO_2_) was monitored using a pulse oximeter (PM 9000 Vet Mindray, Shenzhen, China) with a transmittance probe that was placed on the tongue. Respiratory rate was obtained from the capnograph. Rectal temperature was recorded using a digital thermometer (PM 9000 Vet Mindray, Shenzhen, China) and was maintained between 38.5 and 39.5 °C using a thermal warming blanket (HP300-A HoMedics, Commerce, MI, USA).

Cardiopulmonary variables including HR, SAP, DAP, MAP, Rr and body temperature were recorded during induction of anesthesia, and every 5 min during the maintenance of anesthesia.

Arterial blood samples for gas analysis were collected into 2 mL heparinized syringes, before premedication (refence point) by a direct sample taken from the left auricular artery; then at 15 and 30 min after surgery started; and 10 min after extubation. The last three samples were obtained from the catheterized right auricular artery. Samples were analyzed for oxygen partial pressure (Pp-O_2_), carbon dioxide partial pressure (Pp-CO_2_), pH, bicarbonate ion concentration (HCO_3_), and glucose and lactate concentrations within 5 min of blood collection using a pre-calibrated blood-gas analyzer (i15Vet Edan, Shenzhen, China).

### 2.4. Evaluation of Anesthetic Depth

To maintain surgical anesthesia, the rate of delivery of propofol (in TIVA) or isoflurane (in PIVA) was always adjusted by the same anesthetist to ensure the consistency of the following clinical signs, which were assessed every minute during the entire anesthetic procedure: the absence of palpebral reflex, ventro-medial eye rotation, loss of mandibular and neck muscle tone, absence of purposeful movement in response to surgical stimulation, and minimal changes in the autonomic response (± 20%) variation from baseline values of HR and MAP.

### 2.5. Determinants of Isoflurane Vaporizer Setting and Propofol CRI

Inspired, and end-tidal isoflurane (animals in the PIVA group only), and expired CO_2_ concentrations were measured using an infrared gas-anesthetic agent analyzer and capnograph, respectively, which were included in the multiparameter monitor through a side stream sampling line connected to the proximal end of the endotracheal tube. The gas analyzer was calibrated for CO_2_ and isoflurane before each anesthesia procedure with a standard gas mixture provided by the manufacturer (Mindray DS Calibration Gas canister, Shenzhen, China).

Goats were able to breathe spontaneously throughout the procedure, unless PE’CO_2_ values rose above 60 mmHg for over 5 min, in which case mechanical ventilation was started to maintain eucapnia (35–45 mmHg ET’CO_2_). The ventilation mode delivered by the ventilator was volume-control with initial settings of 10 mL/Kg tidal volume, Rr: 10 rpm and I:E ratio 1:2.5 (Ohmeda 7800, Datex-Ohmeda, Helsinki, Finland).

For animals in the TIVA group, if MAP or HR increased over 20% from baseline the surgery was interrupted and a propofol bolus of 1 mg/kg was administered for over 30 s IV and propofol CRI increased by 0.08 mg/kg/min until HR and MAP returned to values within 20% of the baseline. Conversely, if MAP decreased over 20% of baseline values, then propofol CRI decreased by 0.08 mg/kg/min or until HR and MAP returned to the previously recorded values. If MAP decreased below 60 mmHg a bolus of 5 mL/kg of Hartmann solution was administered IV for 15 min.

For animals in the PIVA group, if MAP or HR increased over 20% of baseline values the surgery was stopped and FE’ISO increased by 0.2% units or until HR and MAP returned to the previously recorded value. The oxygen flow rate increased to 100 mL/kg/min during this process. Conversely, if MAP decreased over 20% of baseline values FE’ISO decreased by 0.2% units or until HR and MAP returned to previously recorded values. If MAP decreased to 60 mmHg or below, Hartmann solution at 5 mL/kg was infused for 15 min.

### 2.6. Surgical Procedure

Surgery started 45 min after the beginning of CRIs and completion of instrumentation. All surgeries were performed through a paramedian incision, always performed by the same surgeons using the technique described by Baird, [25]. Cardiopulmonary variables and anesthetic requirements were recorded immediately at the beginning of the skin incision (T0), immediately after laparotomy (T1), during traction and exteriorization of the abomasum (T2), during abomasotomy (T3), at the midpoint of the closure of the abomasum (T4), at the midpoint of the closure of the abdominal wall (T5), during the closure of the subcutaneous tissue (T6) and at the midpoint of the closure of the skin (T7).

Surgery time (time elapsed from the first incision until the placement of the last suture), anesthesia time (time elapsed from the injection of propofol until turning off the vaporizer or infuser pump), and time to extubation (time elapsed from turning off the vaporizer dial or infuser pump until extubation) were recorded.

Goats were disconnected from the inhalation anesthetic delivery circuit at extubation time, which was adjudged by the return of the swallowing reflex. Time to first head lift, time to attain a sternal position (time elapsed between turning off the vaporizer or infuser pump and the attainment of the sternal position), and time to a standing position (time elapsed between turning off the vaporizer or infuser flow and the attainment of a standing position, which was defined as the ability to remain standing at least 10 s without support) were also documented.

### 2.7. Postanaesthetic Sedation and Recovery

Sedation during recovery was assessed every hour, for up to 4 h after extubation using a 0–3 scale as described above (anesthetic procedure and study design). Quality of recovery from anesthesia was scored using the scale described by Carroll et al. [18] at 0, 15, 30, 45, and 60 min postoperatively, and then every hour till hour 4. This scale includes parameters such as transition to alertness, coordination, behavior, and score of analgesia. Postoperative analgesia was again scored at hour 4, then at 24, 48, 72, and 96 h.

After the last sedation assessment, goats were returned to their housing pen, then a dose of intramuscular meloxicam (0.3 mg/kg; Melodex, Aranda, CDMX, Mexico) was administered every 24 h for 3 consecutive days. If an animal scored 3 in the scale described by Carroll et al. [18], the goat would receive rescue analgesia using intramuscular flunixin meglumine (1.0 mg/kg; Finadyne, MSD Animal Health, Kenilworth NJ, USA) instead of the scheduled meloxicam dose.

### 2.8. Statistical Analysis

Sample size calculation was based on a difference between means (recovery times) of two independent groups, based on a previous pilot study. It was set a mean of 20 ± 5 min for PIVA group and 30 min for TIVA group, at an alpha value of 5% and power of 80%. The calculated sample size was 8 animals per group (Clin calc.com https://clincalc.com/stats/samplesize.aspx accessed on 14 January 2017). A Shapiro–Wilk test was used to test data for distribution patterns.

Nonparametric and ordinal variables are expressed as median and range. Within each group, changes in sedation scores, and quality data over time were analyzed using Friedman tests followed by Dunn’s multiple comparison test, when a significant difference was detected.

Differences in nonparametric data between treatments were analyzed using the Mann-Whitney test. Parametric data are expressed as mean ± standard deviation (SD) and range. Physiological data (HR, SAP, DAP, MAP, rectal temperature, Rr) and blood-gas and analyte data (Pp-O_2_–⁠Pp-CO_2_, HCO_3_, Lactate, pH, glucose) were tested for statistically significant differences over time using ANOVA (Analysis of variance) for repeated measures, followed by Dunnett′s test when appropriate. Unpaired *t*-tests were used for comparisons between treatments at each time point and for the values of anesthesia time, surgery time, time to first head lift, time to accomplish sternal recumbency, and time to standing a. Data were analyzed with a statistical software package (Graphpad, Prism 5.0, San Diego, CA, USA. Differences were considered significant at *p* < 0.05.

## 3. Results

### 3.1. Preanesthetic Sedation and Induction Dose of Propofol

Administration of fentanyl, lidocaine, and ketamine resulted in heavy sedation of all goats. Median scores observed were 3 for both PIVA (range 1–3) and TIVA (range 2–3) protocols (*p* = 0.8512).

Following fentanyl premedication but before induction of anesthesia, abnormal behavioral signs (e.g., exaggerated tail-wagging, chewing movements and restlessness) were observed in goats from both PIVA and TIVA protocols. Mean propofol doses required for induction of anesthesia were 3.78 ± 0.38 mg/kg for both protocols.

### 3.2. Anesthesia Time and the Surgery Time

Mean anesthesia time was 102 ± 15 and 92 ± 16 min for PIVA and TIVA protocols (*p* = 0.2161), respectively. Mean surgery time was 57 ± 9 and 53 ± 6 min for PIVA and TIVA protocols (*p* = 0.3021), respectively. The surgery started 39 ± 5 and 35 ± 6 min after the induction of anesthesia, for PIVA and TIVA protocols respectively (*p* = 0.4639).

### 3.3. Cardiopulmonary Variables

Cardiopulmonary variables did not differ significantly between PIVA and TIVA protocols (Table 1), except for SAP at surgical times T3, T5, and T6; at this time points, significantly higher values were observed during TIVA (*p* = 0.0382, 0.0398 and 0.0143, respectively).

Rectal temperature was maintained within the predetermined range (38.5–39.5 °C) and there were no significant differences between PIVA and TIVA protocols. The concentration of arterial blood gases and analytes did not differ significantly between PIVA and TIVA protocols; however, some differences were observed only in arterial blood gases and pH when comparing references points values (Table 2) to those from minutes 15 and 30, but not of clinical relevance.

### 3.4. Anesthetic Requirements to Achieve an Acceptable Anesthetic Depth

During the surgical procedure, the observed mean FE′Iso was 0.81 ± 0.20% and the mean CRI of propofol was 0.44 ± 0.07 mg/kg/min, (Table 3). Percentage reduction in FE′Iso and ranges when compared with initial FE′Iso (1.2%) were 26.88% (17.6% to 33.92%) (*p* < 0.0001), while percentage increase in CRI of propofol when compared with initial CRI (0.3 mg/kg/min) were 46.9% (36% to 53%) (*p* < 0.0001). There were no statistical differences in either PIVA or TIVA protocols for anesthetic requirements from T0 to T6. All goats showed lack of palpebral and podal reflexes, ventromedial rotation of the eye and relaxed muscular tone in the neck; except for two goats in TIVA, and one goat in PIVA groups during the traction and exteriorization of the abomasum, where palpebral reflex were slight present in those animals.

### 3.5. Anesthesia Recovery

Mean time to extubation was 14.7 ± 5.6 and 15.3 ± 4.8 min for PIVA and TIVA protocols (*p* = 0.8265), respectively. Mean time to first head lift was 18.8 ± 6 and 22.7 ± 10.7 min for PIVA and TIVA protocols (*p* = 0.4501), respectively. Mean time to sternal recumbency was 22.5 ± 6.8 and 27.4 ± 11.9 min for PIVA and TIVA protocols (*p* = 0.3038), respectively.

Mean time to standing was 42.3 ± 14.9 and 44.3 ± 11.7 min for PIVA and TIVA protocols (*p* = 0.7564), respectively. Times to extubation, sternal position, and standing position were not significantly different between TIVA and PIVA protocols.

Median scores measured for recovery quality were significantly lower in PIVA than in TIVA from minutes 30 to 60 (Table 4). Some abnormal behavioral signs, including exaggerated tail-wagging, vocalization, restlessness, ear motions and leaning on objects were observed in TIVA group (*n* = 4) during the recovery period.

### 3.6. Postoperative Sedation and Analgesia Scores

Median postoperative sedation scores were not significantly different between PIVA and TIVA protocols (Table 4). No animal from any group needed rescue analgesia.

## 4. Discussion

The results of the present study have shown that propofol or isoflurane combined with CRIs of fentanyl–lidocaine–ketamine produced a satisfactory quality of anesthesia during surgery, with comparative minimal impact on cardiopulmonary function, but with differences in recovery quality.

In the present study the goats reached deep sedation with half the dose of fentanyl used previously by Dzikiti et al. [26] (20 µg/kg) to reach the same sedation state. Tallarida, [27] and Hendrickx et al. [28]. reported that combinations of two or more drugs may generate interactions that can be categorized as “synergistic”, “additive” or “infra-additive”, when their combined effect exceeds, equals, or is less than that of the total sum of the effects of the individual drugs. We therefore postulate that the combination of these three drugs may generate an additive effect, which may explain the observed deep sedation of goats with a considerably lower dose of fentanyl.

In the present study, we observed a series of abnormal behaviors during anesthetic premedication with fentanyl. These behavioral changes have been reported in association with the administration of opioids in ruminants, deriving from central nervous system stimulation [8,29,30]; changes such as an increase in vocalization and agitation [31], chewing movements and nystagmus [32], and excessive tail-wagging [21] have been reported. These changes are not common when opioids are administered with a sedative or anesthetics agents, or when administered to animals in pain [4]. This may explain our observation that excitatory behavioral effects were (e.g., exaggerated tail-wagging, chewing movements and restlessness) less pronounced once the lidocaine and ketamine were administered after fentanyl.

The use of sedative drugs facilitates restraint during the induction of anesthesia and reduces drug requirements for induction anesthesia and maintenance [24]. The mean propofol dose for anesthesia induction in the present study was 3.9 mg/kg, which is considerably lower than the doses reported in previous studies with non-premedicated goats (e.g., 5.1 mg/kg, [33]; and 5.3 mg/kg, [34]). The use of low dose propofol, can decrease the presence of side effects associated with the use of propofol during induction of anesthesia [15,33]. Moreover, myoclonus, a reported adverse effect associated with the use of propofol during induction of anesthesia [15,33,34], was not observed in the present study, which highlights the safety of our anesthesia induction protocol using this drug.

Besides the premedication or co-administration with other nervous system depressant drugs such as fentanyl in goats [26], other factors not necessarily related to drugs, such as the temperament of goats, can influence the effective dose for induction of anesthesia. Lower doses of propofol for induction of anesthesia (e.g., 3.0 mg/kg) have been reported in pet goats, which are expected to be more docile or accustomed to being handled by humans [33,35]. The goats included in this study did not have a period of adaptation and were anaesthetized for the first time at the moment of the abomasotomy; still, the propofol dose required for induction of anesthesia was low.

It should be noted that in the present study, the mean propofol infusion rate required for effective maintenance of anesthesia (0.44 mg/kg/min) was higher than in previous studies on premedicated goats. Larenza et al. [36] reported propofol infusion rates of 0.2 mg/kg/min and Dzikiti et al. [26]. reported infusion rates of 0.3 mg/kg/min. In both studies, the reduction of the propofol infusion rate required for maintenance of anesthesia was the result of administration of fentanyl (0.02 mg/kg/h) combined with midazolam (0.3 mg/kg/h) [26], or of ketamine alone (0.03 mg/kg/min) [36].

The above dissimilarities on infusion rates may be the result of differences in the noxious stimulus used in above studies. In those reports [26,36], animals were subjected to supramaximal noxious stimulus, while in the current study animals were subjected to a surgical procedure and this stimulus may have exceeded the standardized supramaximal stimulus applied in research studies [20], the differences in noxious stimulation may have a great effect on the required infusion rate of anesthetic drugs [37]. Surgical stimulation may require higher anesthetic drug infusion rates in comparison with other methods used for imitating noxious stimulation [38].

Recently, Vieitez et al. [20] reported a propofol infusion rate of 0.2 mg/kg/min in one goat undergoing craniotomy, during co-administration of a CRI of lidocaine (50 μg/kg/min), midazolam (0.15 mg/kg/h) and fentanyl (6 μg/kg/h). This propofol infusion rate was lower than the mean propofol infusion rate obtained in the current study. Even when a cranial surgical procedure was performed in that case, it should be noticed that the goat had neurological abnormalities such as disorientation, inability to run and scape and diminished alert mentation as a result of cerebral cyst; furthermore, this goat received premedication with xylazine and morphine, drugs that may have reduced the requirements of general anesthesia [30].

In the same way, the pain associated with abomasotomy has not yet been characterized in ruminants. Nevertheless, in humans [39] caesarean section, which sometimes implies traction, but not always exteriorization of the uterus, and gastrectomy among other abdominal surgical procedures, were correlated with higher pain scores than skull and/or brain surgery.

Considering the above statements and the results of the present study, we could speculate that abomasotomy is considerably a painful intervention which may explain the higher propofol infusion rate needed in the present study for effective anesthesia. During painful procedures (such as abomasotomy) propofol lacks any pain-relieving effects, so higher infusion rates are needed in combination with analgesic drugs [15,16].

Studies by different authors have reported MAC (minimum alveolar concentration) isoflurane values in goats ranging from 1.23% to 1.50% [40,41,42]; the mean FE′Iso observed in the present study (0.81%) is much lower. These differences may be explained by the possible additive effect of the combination of the three drugs used in the present study, as reported in dogs by Aguado et al. [23], these authors reported a reduction of up to 97% in isoflurane anesthetic requirements, relative to MAC of isoflurane, in bitches undergoing ovariohysterectomy when lidocaine, ketamine, and fentanyl were used in combination. In the present study, the time elapsed between induction and T0 may have assured a steady-state concentrations of CRI analgesics enough for decreasing the requirements of FE’Iso, showing a greater sparing effect at the beginning of the surgery.

Increased HR values observed over time (Table 1) may be the result of surgical stimulation and the subsequent activation of the sympathetic nervous system during pulling and exteriorization of the abomasum (T2) and its subsequent reinsertion (T4). During surgery, SAP values were significantly lower in PIVA than in TIVA at T3, T5, and T6. This difference may be explained by the fact that isoflurane reduces vascular resistance following vasodilatation, thereby decreasing blood pressure [42]. However, SAP values in animals in the PIVA group were never lower than those at the beginning of surgery (T0), which means that the requirements for isoflurane anesthesia were adequate during the surgical process since dose-related major cardiovascular depressive effects, were not observed; nevertheless, a slight increase of HR values, along with subsequent increase of blood pressure were recorded up to T3–T4. Conversely, at these time-point HR values increased but blood pressure values decreased. It is likely that this effect is due to the increase in FE’Iso during T3 and T4 (Table 3).

As indicated by the average Pp-CO_2_ values recorded, no notable respiratory depression was observed in either TIVA or PIVA protocols in goats performing spontaneous respiration. In anaesthetized goats, lung ventilation may decrease, and hypercapnia is common when spontaneous breathing is preserved [36]. Goats in the TIVA group had higher Rr, although no improvement was observed in ventilation, relative to the value of Pp-CO_2_ and Pp-O_2_. Goats in the PIVA group had Pp-CO_2_ values that were slightly lower than those of animals in the TIVA group; however, Pp-CO_2_ values were within a clinically acceptable range during anesthesia in both groups. On the other hand, a similar degree of respiratory acidosis occurred in both groups due to an increase in Pp-CO_2_, although there were no significant differences between them.

As indicated by the median quality scores, recovery from anesthesia in the animals in the PIVA group was smooth and uneventful (signaling high-quality recovery), which is in keeping with a previous report on goats anaesthetized with isoflurane and fentanyl [21].

In goats, recovery from isoflurane anesthesia is expected to be rapid [21,42], especially when isoflurane is used at low concentrations, as in the present study. In contrast, in animals of the TIVA group, recovery was characterized by abnormal behavioral signs in four goats (e.g., exaggerated tail-wagging, vocalization, restlessness), and by a higher degree of ataxia, which resulted in a higher number of attempts to stand up. These behavioral changes are often associated with the administration of opioids [24,30,31], and are consistent with a report by Dzikiti et al. [26] who indicates that care should be taken during recovery from anesthesia induced by a combination of propofol and fentanyl, since excitatory behavioral signs may be expected. These authors [26] also suggest that stopping fentanyl infusion earlier may minimize the occurrence of excitatory effects during recovery from anesthesia.

Some limitations of this study included the lack of more treated groups to elucidate the sparing effect of each drug separately; also, in our study design we managed to use fixed CRI doses of fentanyl, lidocaine, and ketamine that may not be always clinically applicable. In addition, in the present study we only included females, adults, and tropical creole goats.

Before the present study, no reports had described the quality of recovery from anesthesia from TIVA performed using propofol, fentanyl, lidocaine, and ketamine in goats. This information is fundamental for post-surgical aiming at high-quality recovery that is not limited to the choice of an anesthesia protocol. Good recovery quality is essential for safety and welfare of the patient.

## 5. Conclusions

Propofol or isoflurane, used in TIVA or PIVA protocols respectively, combined with CRIs of fentanyl–lidocaine–ketamine produced a satisfactory quality of anesthesia during surgery with minimal impact on cardiopulmonary function. Nevertheless, recovery after anesthesia induced by the combination of propofol fentanyl–lidocaine–ketamine might be of poor quality.

## Figures and Tables

**Table 1:** Cardiopulmonary variables (mean ± SD) observed in goats undergoing abomasotomy and receiving continuous infusion of fentanyl (0.01 mg/kg/h), lidocaine (0.05 mg/kg/min) and ketamine (0.05 mg/kg/min) in conjunction with isoflurane (PIVA) or continuous infusion of propofol (TIVA) for anesthetic maintenance.

**Variables**	**Treatment**	**Time Points**								
		**Reference Point**	**T0 (Baseline)**	**T1**	**T2**	**T3**	**T4**	**T5**	**T6**	**T7**
HR	TIVA	70 ± 9.4	65 ± 19.7	69 ± 17.5	78 ± 16.1†	75 ± 18.1	77 ± 20.2 †	80 ± 17.7 †	79 ± 17.4 †	76 ± 17.6 †
	PIVA	66 ± 9.9	84 ± 19.3 *	86 ± 18.9 *	88 ± 15.6 *	92 ± 16.1 *	95 ± 18.6 * †	89 ± 21 *	88 ± 18.7 *	88 ± 17.5 *
fR	TIVA	21 ± 5.0	11 ± 4.8 *	12 ± 5.4 *	16 ± 6.8 *	12 ± 4.1 *	14 ± 4.8 *	12 ± 4.7 *	15 ± 6.6 *	15 ± 5.7 *
	PIVA	21 ± 5.3	10 ± 3.1 *	9 ± 3.5 *	12 ± 5.8 *	11 ± 3.2 *	11 ± 4.4 *	10 ± 4.2 *	11 ± 5.8 *	14 ± 6 * †
SpO2	TIVA	98 ± 1.8	97 ± 2.6	97 ± 2	96 ± 2.9	97 ± 1.4	97 ± 2.1	98 ± 0.9	98 ± 1	98 ± 0.8
	PIVA	96 ± 2.3	97 ± 1.2	97 ± 1.5	97 ± 1.5	97 ± 1.8	97 ± 2.1	97 ± 1.8	98 ± 0.7	98 ± 0.7
ET’CO_2_ (mmHg)	TIVA	41.3 ± 2.8	40.3 ± 3.5	41.3 ± 4.6	41 ± 5.8	42.3 ± 4.5	40.5 ± 5.5	40.4 ± 5.1	39.7 ± 3.5	39.4 ± 4
	PIVA	42 ± 2.6	40.7 ± 5.2	40.7 ± 5.7	41.2 ± 8.1	40.6 ± 4.2	40 ± 7.4	41 ± 6.3	38.2 ± 47	35.8 ± 6.5
SAP (mmHg)	TIVA	134 ± 24.5	98 ± 7.7 *	109 ± 8.9 *†	129 ± 15†	118 ± 14 ^a^*†	110 ± 10 * †	119 ± 10 ^a^*†	114 ± 6 ^a^ * †	110 ± 7.6 * †
	PIVA	137 ± 26.2	97 ± 10.8 *	104 ± 12 *	124 ± 21.3†	105 ± 9.4 ^b^*	104 ± 12.8 *	107 ± 12.4 ^b^*	103 ± 10.2 ^b^*	107 ± 12.5 *
DAP (mmHg)	TIVA	85 ± 25.3	72 ± 12.7 *	81.5 ± 11.6 †	99 ± 12.4 * †	88 ± 12.3 †	84 ± 12†	95 ± 9.8 †	87 ± 8.9†	85 ± 9.2 †
	PIVA	94 ± 21.3	73 ± 7.8 *	78 ± 10.6 *	94 ± 17.5 †	81 ± 11.3 *	80 ± 13.9 *	84 ± 13.2 †	81 ± 11.7 *	86 ± 11.1 †
MAP (mmHg)	TIVA	102 ± 22.7	80 ± 11.3 *	90 ± 10.1 †	108 ± 12.5 †	96 ± 13.9 †	92 ± 11.3 †	101 ± 9.6 †	97 ± 8.5†	93 ± 8.4 †
	PIVA	100 ± 10	80 ± 10.7 *	86 ± 10.1 *	104 ± 18.6 †	88 ± 10.6 *	89 ± 12.6 *	91 ± 12.3 †	89 ± 10.2 *	92 ± 12.1 * †

Heart rate (HR), respiratory rate (Rr), hemoglobin oxygen saturation (SpO_2_), end-tidal CO_2_ (ET-CO_2_), systolic arterial pressure (SAP), diastolic arterial pressure (DAP) and mean arterial pressure (MAP). * significantly different within the same group compared to reference point. † significantly different within the same group compared to the value of T0 (baseline). ^a,b^ significantly different between groups (*p* < 0.05).

**Table 2:** pH and blood-gas values (mean ± SD) observed in goats undergoing abomasotomy and receiving continuous infusion of fentanyl (0.01 mg/kg/h), lidocaine (0.05 mg/kg/min) and ketamine (0.05 mg/kg/min) in conjunction with isoflurane (PIVA) or continuous infusion of propofol (TIVA) for anesthetic maintenance.

Variable	Treatment	Reference Point	15	30	POST
pH	TIVA	7.529 ± 0.03	7.36 ± 0.04 *	7.38 ± 0.04 *	7.449 ± 0.05
	PIVA	7.498 ± 0.01	7.378 ± 0.03	7.393 ± 0.08	7.466 ± 0.04
Pp-O_2_ (mmHg)	TIVA	84.3 ± 4.04	493.7 ± 177.8*	522 ± 129.6 *	98 ± 4.35
	PIVA	88 ± 2.6	502.7 ± 12.64*	582.3 ± 100.1 *	95.67 ± 7.23
Pp-CO_2_ (mmHg)	TIVA	37.6 ± 2.17	46.8 ± 1.01*	44.63 ± 1.38 *	39.4 ± 1.38
	PIVA	37.83 ± 6.9	44.27 ± 2.37*	43.3 ± 2.37 *	41.5 ± 1.7
Glucose (mg dL)	TIVA	51 ± 10.58	55.33 ± 3.21	51 ± 6.92	45 ± 6
	PIVA	41 ± 7.55	46.33 ± 15.5	37.33 ± 7.76	44.67 ± 8.96
Lactate (mmol L)	TIVA	0.68 ± 0.61	0.3 ± 0	0.3 ± 0	0.38 ± 0.13
	PIVA	0.39 ± 0.10	0.3 ± 0	0.3 ± 0	1.13 ± 1.44
HCO3- (mmol L)	TIVA	30.77 ± 3.48	30.3 ± 3.30	30.33 ± 4.13	26.57 ± 2.17
	PIVA	28.57 ± 4.08	31.9 ± 4.38	32.5 ± 3.95	29.37 ± 3.55

Oxygen partial pressure (Pp-O2), carbon dioxide partial pressure (Pp-CO2), bicarbonate ion concentration (HCO3). * significantly different within the same group compared to reference point (*p* < 0.05).

**Table 3:** FE’Iso (%) (mean ± SD) of isoflurane (PIVA) and milligrams (mg/kg/minute) (mean ± SD) administered during constant propofol infusion (TIVA), observed in goats undergoing abomasotomy and receiving continuous infusion of fentanyl (0.01 mg/kg/h), lidocaine (0.05 mg/kg/min) and ketamine (0.05 mg/kg/min).

Treatment	Variable	Time Points							
		T0	T1	T2	T3	T4	T5	T6	T7
PIVA	FE’Iso (%)	0.76 ± 0.20	0.77 ± 0.18	0.78 ± 0.16	0.87 ± 0.19	0.86 ± 0.22	0.87 ± 0.24	0.79 ± 0.22	0.77 ± 0.19
TIVA	mg/kg/min	0.42 ± 0.05	0.44 ± 0.06	0.45 ± 0.06	0.44 ± 0.05	0.44 ± 0.08	0.44 ± 0.07	0.45 ± 0.08	0.40 ± 0.08

**Table 4:** Median and range values of postoperative sedation, recovery quality, and analgesia scores, observed in goats that underwent abomasotomy and received continuous infusion of fentanyl (10 μg/kg/h), lidocaine (50 μg/kg/min) and ketamine (50 μg/kg/min) in conjunction with isoflurane (PIVA) or continuous infusion of propofol (TIVA) for anesthetic maintenance.

Variable	Treatment	Time											
		0 min	15 min	30 min	45 min	60 min	120 min	180 min	240 min	24 h	48 h	72 h	96 h
Sedation	PIVA	3 (3–3)	3 (2–3)	2 (2–2)	1 (1–2)	1 (0–1)	0 (0–1)	0 (0–1)	0 (0–0)	-	-	-	-
	TIVA	3 (3–3)	3 (2–3)	3 (1–3)	2 (0–2)	1 (0–1)	1 (0–1)	1 (0–1)	0 (0–1)	-	-	-	-
Recovery quality	PIVA	3 (3–3)	3 (2–3)	2 ^a*^ (2–3)	2 ^a**^ (1–2)	1 ^a***^ (1–1)	1 (1–1)	1 (1–1)	1 (1–1)	-	-	-	-
	TIVA	3 (3–3)	3 (3–3)	3 ^b^ (3–3)	2 ^b^ (2–3)	2 ^b^ (2–2)	1 (1–2)	1 (1–1)	1 (1–1)	-	-	-	-
Analgesia	TIVA	1 (1–1)	-	-	-	-	-	-	2 (1–2)	2 (1–2)	2 (1–2)	1 (1–1)	1 (1–1)
	PIVA	1 (1–1)	-	-	-	-	-	-	2 (1–2)	2 (1–2)	2 (1–2)	1 (1–1)	1 (1–1)

^a,b^ significantly different between groups (*p* < 0.05). * *p* = 0.0003, ** *p* = 0.004, *** *p* < 0.0001. - No measurement was made.

## Data Availability

Not applicable.
